# Structure, (governance) and health: an unsolicited response

**DOI:** 10.1186/1472-698X-6-12

**Published:** 2006-09-15

**Authors:** Daniel D Reidpath, Pascale Allotey

**Affiliations:** 1 Centre for Public Health Research, Brunei University, West London, UK

## Abstract

**Background:**

In a recently published article, it was suggested that governance was *the *significant structural factor affecting the epidemiology of HIV. This suggestion was made notwithstanding the observed weak correlation between governance and HIV prevalence (*r *= .2). Unfortunately, the paper raised but left unexamined the potentially more important questions about the relationship between the broader health of populations and structural factors such as the national economy and physical infrastructure.

**Methods:**

Utilizing substantially the same data sources as the original article, the relationship between population health (healthy life expectancy) and three structural factors (access to improved water, GDP per capita, and governance) were examined in each of 176 countries.

**Results:**

Governance was found to be significantly correlated with population health, as were GDP per capita, and access to improved water. They were also found to be significantly correlated with each other.

**Conclusion:**

The findings are discussed with reference to the growing interest in structural factors as an explanation for population health outcomes, and the relatively weak relationship between governance and HIV prevalence.

## Background

The work of Sen and others on human capabilities [1,2] has provided an important framework for the exploration of the relationship between structure, capabilities, and health [3-6]. Within this framework structural factors (which include physical infrastructure and the wealth of a society, through to the political and policy environment) can be seen to create a continuum of effects which may enhance or diminish the health of populations. A ready example of this effect can be observed in individuals who have paraplegia, whereby in one socio-cultural context the impact of paraplegia is amplified by the lack of physical infrastructure, the lack of care, and an unforgiving social milieu; whereas in a more forgiving context the reverse is true [7].

HIV provides another example of the relationship between health, structure and capabilities. It is structural factors that affect which groups in the population are most vulnerable to infection, and by extension, the probability that any one individual will become infected [8-10]. Structural factors also affect the impact of the virus on individuals who are already infected, by decreasing access to social goods and resources such as healthcare, education, and employment [11,12]. Thus, structure mediates individual vulnerability and the impact on those who are already infected.

The latest research to examine structural issues and HIV was published recently in this journal, and focussed on governance [13], which increasingly is argued to be important to health generally, and HIV in particular [14,15]. The study by Menon-Johansson utilized global data, and correlated a series of six *World Bank *measures of governance [16] with the national prevalence of HIV in 149 countries. Although this was not the first research of its kind [15], the use of the *World Bank *measures of governance added a new and important dimension. It expanded the consideration of structural factors from the very concrete, physical environment, or the readily measured policy environment (wealth, literacy, and unemployment), through to the very abstract, and it did it with this new and increasingly accepted empirical measure. While the data, sourced from the *World Bank *and the UN are less than perfect, they provide an opportunity for new ways of looking at relationships between structure and health.

Notwithstanding its innovation, the paper was surprising on a number of levels. First, the identified relationship between the level of governance and the prevalence of HIV was weak. The correlation between any of the six measures and HIV prevalence never exceeded .20 and was as low as .16. In other words, the measures of governance could not account for more than 4% of the variance of HIV prevalence. This finding is, in some ways, contrary to expectation given the kind of interest that Sen's work on capabilities has generated, and it raises questions about the value of the approach to issues of population health. In spite of the weakness of the relationship, much of Menon-Johansson's paper was devoted to explaining why governance was important to, and by implication causally related to, the prevalence of HIV. This was in spite of the author's careful observance of the rule that one should not ascribe causation when an analysis can only demonstrate association. The conclusion was ambiguously clear:

"HIV prevalence is significantly associated with poor governance. International public health programs need to address societal structures in order to create strong foundations upon which effective healthcare interventions can be implemented." [13]

The capabilities approach, however, necessarily invites a broader consideration of structural factors that may affect health beyond simply governance. Tantalizingly, Menon-Johansson introduced data on other structural factors such as access to water and countries' per capita wealth. Unfortunately these data were not included in the analysis of HIV prevalence. Readers are left with a troubling disjunction between the rhetorical importance of structure and a statistically significant, but largely unimpressive association between the level of governance and HIV prevalence. A re-analysis of the data is required, looking at the relationship between structural factors (including governance) and a broader measure of population health.

## Methods

The analysis is based on the types of data described by Menon-Johansson in the original paper, and made available as a supplementary electronic file [17]. As previously noted, there are concerns with the quality of these data. However, they remain the best available country-level estimates – a point to which we return in the Discussion. The focus here is on the interplay between population health outcomes and the structural factors including governance, which may be identified in the data and operate at a country-level. These are operationalized below.

### Population health measures

Two principle measures of population health were examined. "Healthy Life Expectancy" (HALE) was used instead of Menon-Johansson's choice of life expectancy, because HALE also takes account of the loss of health associated with morbidity [18]. Estimates for 2002 were obtained from the World Health Organization [19]. The infant mortality ratio (IMR) of infant deaths per 1000 live births was also used. IMR has a long history as a proxy measure of a population's health and continues to have value [20]. The IMR estimates for 2002 were obtained from the *World Health Report 2004 *[21].

### Structural factors

Three qualitatively distinct structural factors were adapted from Menon-Johansson's paper for re-analysis here: governance, gross national product (GDP) per capita, and access to water. Governance is an abstract notion of the manner in which countries function. GDP per capita is a measure of a population's wealth, the value of which is ultimately only realized through the purchase of goods or services. Access to water is an indicator of a country's basic physical infrastructure.

Following Menon-Johnansson, data on six separate measure of governance for 2002 were obtained from the *World Bank *web site [22]. In developing governance indicators, the World Bank broadly defined governance "... as the traditions and institutions by which authority in a country is exercised" (p.2) [16]. Within this framework, governance is treated as a six-dimensional construct comprising (pp.3–4):

1. Voice and accountability;

2. Political stability;

3. Government effectiveness;

4. Regulatory quality;

5. Rule of law; and

6. Control of corruption.

A principal components analysis of the data was conducted with a view to reducing the number of dimensions along which governance was measured [23]. The analysis showed that the six measures could be successfully reduced to a single composite measure that accounted for the majority of the available variance – 87%. In measurement terms there was little justification for retaining six measures when one would suffice. This also highlights subjective assumptions about the dimensionality of governance, but nonetheless supports the general thrust of this commonsense notion – another point to which we return in the Discussion.

A composite measure of governance was created from the first principal component [23]. For interpretive convenience, the governance scores were rescaled to lie in the interval 0 (low governance) to 100 (high governance). To provide a sense of these data, Table 1 shows those countries with governance scores in the lowest (≤ 10), middle (45–54), and highest (≥ 90) range.

**Table 1:** Countries with low, moderate, or high governance scores.

**Low (0–10)**	**Moderate (45–54)**	**High (90–100)**
Somalia (0)	Turkey (45.7)	Ireland (90.2)
Iraq (3)	Brazil (45.9)	Germany (90.2)
Congo, Dem. Rep. (5.1)	India (46)	Australia (93.2)
Myanmar (7.9)	Romania (46)	Austria (93.6)
Afghanistan (8.2)	Mexico (48.7)	Canada (93.6)
Liberia (8.7)	Morocco (49)	United Kingdom (94)
	Belize (50.5)	Norway (94.4)
	Cape Verde (50.8)	Singapore (95.1)
	Panama (50.9)	Sweden (95.5)
	Sri Lanka (51.1)	New Zealand (95.7)
	Bhutan (51.4)	Iceland (96)
	Saudi Arabia (52.0)	Netherlands (96.7)
	Seychelles (52.0)	Denmark (96.9)
	Mongolia (52.5)	Switzerland (97.9)
	Bulgaria (52.9)	Finland (99.4)
	Croatia (53.2)	Luxembourg (100)
	Jordan (54.0)	
	Thailand (54.2)	
	Tunisia (54.3)	

The results are as one might anticipate, with conflict ravaged countries like Somalia, Afghanistan, and Iraq appearing among the countries with the lowest governance scores, and countries like Switzerland, Sweden, and Singapore appearing among the countries with the highest scores.

Data on GDP per capita were obtained for each country from the *World Development Indicators 2005 *[24]. The GDP measure used here had been converted to current international dollars using purchasing power parity rates. An international dollar has the same purchasing power over GDP as the U.S. dollar has in the United States.

Access to water was defined in terms of the percentage of the population who have access to "improved water", that is, "... at least 20 litres [of water] per person per day from a source within one kilometre of the user's dwelling ..." (pp. 77–78) [25]. Bottled water was explicitly excluded from the definition of improved water. [21].

Table 2 provides summary information about the selected global measures of population health and the three socio-structural measures that were investigated.

**Table 2:** Summary details of the two population health measures (HALE and IMR) and the three structural factors (governance, GDP, and water) that were examined.

**Measure**	**N**	**Mean**	**SD**	**Min**	**Max**
HALE	176	57.2	11.4	28.6	75
IMR	176	45.1	42.3	3	189
Governance	176	47.5	24.5	0	100
GDP	155	9151	10169	516	60025
Water	138	81.1	19.6	22	100

## Results

The relationships between population health on the one hand, and governance, GDP per capita, and access to improved water, on the other, were each examined in turn.

### Governance

**Figure 1:**
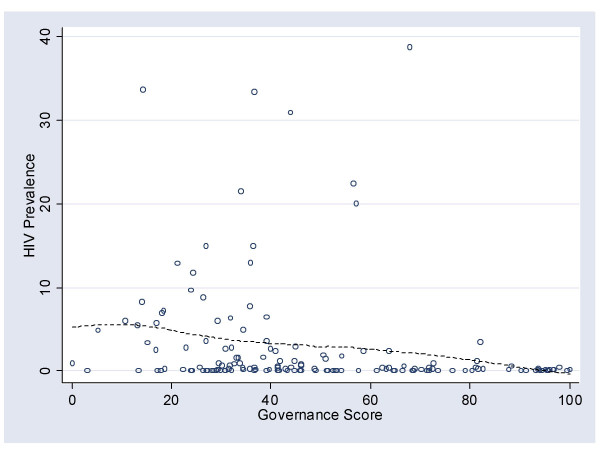
The relationship between the governance score and HIV prevalence.

For comparative purposes a scatterplot of the relationship between HIV prevalence and governance was created (Figure 1). The strength of the relationship between HIV prevalence and governance was very close to that reported by Menon-Johansson (*r *= .2, *p *< .05). A locally weighted regression line (lowess [26]) was also added to the plot, which confirms the weak, variable nature of the relationship between governance and HIV prevalence.

**Figure 2:**
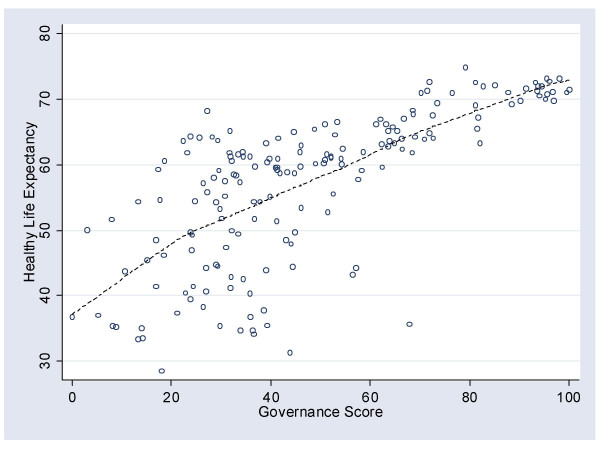
The relationship between the governance score and Healthy Life Expectancy.

The substantive question – the nature of the relationship between governance and population health – was illustrated with a scatterplot between healthy life expectancy (HALE) and governance in each of 176 countries (Figure 2). Again, a lowess line was added to the plot. In contrast to the relationship with HIV prevalence, the correlation between governance and HALE was high *r *= .72, *p *< .001), indicating a substantially stronger relationship than might otherwise have been anticipated. The lowess line shows a basically straight-line relationship between improving governance and improving health.

**Figure 3:**
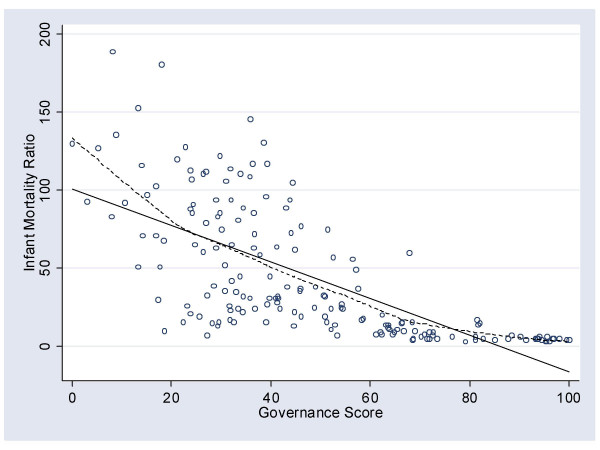
The relationship between the governance score and the infant mortality ratio.

The relationship between governance and the other measure of population health was examined in a similar fashion (Figure 3). The correlation between governance and IMR was again high (*r *= -.68). In addition to the lowess line (dashed), a straight line of best fit was also included (solid). Consistent with the relationship observed between HALE and governance, as governance improved so there was an associated decrease in the IMR. In this case, however, the relationship was a curvilinear one, with increases in governance scores above ~80 associated with IMR asymptoting towards 0.

### GDP per capita

**Figure 4:**
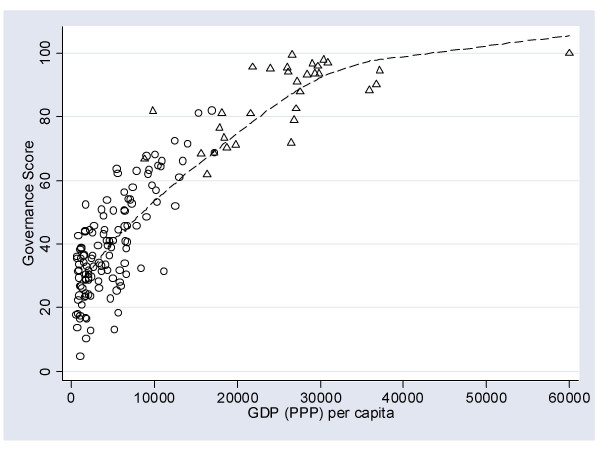
The relationship between GDP per capita and the governance score.

It is undisputed that a country's per capita wealth is strongly associated with population health [27]; and this was confirmed here by the correlation between GDP per capita and HALE (*r *= .69). One might also imagine that the quality of a country's governance is strongly related to a country's per capita wealth – because good governance can itself be a costly exercise. Figure 4 illustrates the relationship between governance and GDP per capita; the lowess line shows a curvilinear fit, with governance sharply improving as national wealth increases (*r *= .88).

Those countries in the top quintile of healthy life expectancy (i.e., countries with a HALE > 67) are also marked on the figure as hollow triangles. That HALE, governance, and GDP per capita are all highly correlated affects the degree to which inferences may be drawn about the independence of any contribution that the structural factors may have on population health. A sequence of regression analyses was conducted to control for this. It showed that governance and GDP per capita were each independently and significantly associated with population health (Table 3). The shared variance was substantial, indicated by the small increase in *R*^2 ^– the variance accounted for by the model – from around 0.5 for the models containing only governance (Model 1) or GDP (Model 2) to 0.52 when combined in Model 3.

**Table 3:** Three progressive models of population health (HALE) exploring the interrelationship of governance (Model 1), GDP (Model 2), and governance and GDP (Model 3).

	**Governance**	**GDP per Capita**	**R**^2^
	
Model 1	0.33 **		0.498
Model 2		.00078 **	0.475
Model 3	0.2 **	.00037 *	0.519

* p < .005 ** p < .001

This goes to the heart of Menon-Johansson's conclusion that governance is an important part of international public health [13]. The relationship between governance and health is at once, potentially more important than that indicated by the HIV data and more complex than that suggested by the paper's conclusion.

### Improved Water

**Figure 5:**
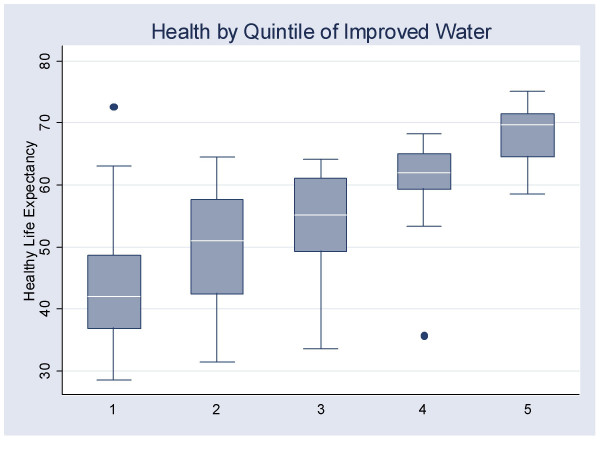
The relationship between access to improved water and healthy life expectancy.

Finally, the relationship between improved water and healthy life expectancy was examined. Countries were first divided into quintiles according to the percentage of the population that had access to improved water. Figure 5 shows a box-plot of the healthy life expectancy of countries in each quintile of water quality.

There was a monotonically increasing relationship between access to improved water and HALE. The median HALE for countries in the poorest quintile of water quality was 42.1, rising steadily to the highest quintile with a median HALE of 61.1. A test for trend showed the improvement in median HALE scores across the quintiles to be significant (*z *= 8.80, *p *< .001) [28].

Thus, all the socio-structural factors from the most abstract (governance) through to the most concrete (access to improved water) were associated with the measures of population health. Furthermore, they were also significantly correlated with each other (Table 4).

**Table 4:** The lower half triangular matrix intercorrelations between the two population health measures (HALE and IMR) and the three structural factors (governance, GDP, and water) that were examined.

**Measure**	HALE	IMR	Governance	GDP	Water
HALE	1				
IMR	-.94	1			
Governance	.72	-.68	1		
GDP	.69	-.63	.88	1	
Water	.67	-.73	.58	0.54	1

An alternative strategy may have been to examine the relationship between health and governance within countries with similar levels of economic development. The quality of the data, unfortunately do not support the analysis.

## Discussion

The data suggest that structure has a powerful effect on population health. Physical infrastructure such the availability of improved water (*r *= .67), economic factors such as GDP per capita (*r *= .69), and quite abstract considerations such as governance (*r *= .72) are all associated with population health. These factors are also correlated with each other, which means that the causal interplay between them is not readily disentangled. In spite of this, at a conceptual level it is easy to see how they might come to influence each other and ultimately the health of populations.

In this regard, great architecture provides a useful analogy. Its creation relies on a complex interrelationship between the design (at the most abstract level), the engineering, and the construction (at the most concrete level). They provide interdependent reinforcement. Excellent design relies on the available materials and the capacity of the builders to take advantage of the materials' qualities. Construction requires good engineering to ensure stability; and functional engineering relies on excellent design. An over reliance on one aspect of design, engineering, or construction to deliver great architecture, is likely to deliver disappointment. It is similarly the case with the interrelationship between structural factors and their capacity to deliver the healthy, functional society. Governance can support broad wealth creation just as wealth supports good governance. Both rely on the existence of sound infrastructure and, in turn, support the development of that infrastructure. For a population to be well educated, well-fed, and generally healthy requires that those interdependent structural features are available, which then supports the entire vision.

The disparity between the specific finding of Menon-Johansson of a weak association between HIV prevalence and governance, and the finding here of a relatively strong association between structure and population health should, on reflection, come as no surprise. Structural measures are likely to show weaker associations with single diseases than they are with broad measures of population health, unless the diseases are ubiquitous. This is because a measure of population health averages the impacts of all the different (rare and common) health conditions. Chagas disease occurs in Brazil but not in Ghana; conversely, guinea worm occurs in Ghana but not in Brazil. To focus on the single disease hides the common structural features that affect exposure to and the severity of diseases in different contexts (e.g., [29]). However, when the health impact of the all diseases are averaged, common themes from the concrete to the abstract may emerge that begin to explain the variation and distribution of poor health.

A clear limitation of both this study and that of the original study by Menon-Johansson is the reliance on country level data on health and structure compiled by the multilateral agencies. The data are often incomplete or rely on imputed values. Furthermore, the extent to which the unidimensional measure of governance adopted here (or the original six dimensional measure), truly captures those abstract structural features of a country will require further investigation to determine it relevance to policy.

It is important that one does not look to single conditions to justify global health policy. It is similarly important that one does not look to a single structural explanation for a population health panacea.

## Conclusion

The 2005 G8 summit in Gleneagles, Scotland recently concluded. Poverty reduction was high on the agenda, as too was the need to improve governance in developing countries. Both, it was suggested, were necessary for improving the health and wellbeing of the populations [30]. The results here suggest that this view is broadly correct. However, and without in anyway diverting this desperately needed change in global politics, a single minded view about which structural factor should be the target of intervention needs to be gently reined. The interrelationship between structure and health, is not straightforward and caution should be exercised before an over reliance develops on the identification of any one element as the panacea for global health. Broad policy approaches informed by the available data should be adopted. They should also be evaluated and judged scrupulously so that as (contextually sensitive) evidence builds, approaches can be changed or altered.

The association between HIV prevalence and governance is weak. The same, however, is not true of the association between governance and the health of populations. Healthy populations tend to have better governance, better physical infrastructure, and greater wealth. The relationship is complicated by the fact that these factors are also correlated with each other. To focus on "governance" as a structural solution to population health would, thus, appear inadequate. Broader approaches need to be adopted which can be refined through appropriate research and evaluation.

## Authors' contributions

DDR performed the analyses, and contributed to the conceptualisation and writing of the paper. PA contributed to the conceptualisation and writing of the paper.

## Pre-publication history

The pre-publication history for this paper can be accessed here:


